# Learning exceptions to category rules varies across the menstrual cycle

**DOI:** 10.1038/s41598-023-48628-x

**Published:** 2023-12-12

**Authors:** Mateja Perović, Emily M. Heffernan, Gillian Einstein, Michael L. Mack

**Affiliations:** 1 Department of Psychology, University of Toronto, 100 St. George St., Toronto, ON M5S 3J3 Canada; 2 Dalla Lana School of Public Health, University of Toronto, Toronto, Canada; 3 Tema Genus, Linköping University, Linköping, Sweden; 4 Rotman Research Institute, Baycrest Hospital, Toronto, Canada

**Keywords:** Cognitive neuroscience, Learning and memory

## Abstract

Ways in which ovarian hormones affect cognition have been long overlooked despite strong evidence of their effects on the brain. To address this gap, we study performance on a rule-plus-exception category learning task, a complex task that requires careful coordination of core cognitive mechanisms, across the menstrual cycle (N = 171). Results show that the menstrual cycle distinctly affects exception learning in a manner that parallels the typical rise and fall of estradiol across the cycle. Participants in their high estradiol phase outperform participants in their low estradiol phase and demonstrate more rapid learning of exceptions than a male comparison group. A likely mechanism underlying this effect is estradiol’s impact on pattern separation and completion pathways in the hippocampus. These results provide novel evidence for the effects of the menstrual cycle on category learning, and underscore the importance of considering female sex-related variables in cognitive neuroscience research.

## Introduction

Despite efforts to reduce the longstanding sex bias in psychology and neuroscience research, the effects of ovarian hormones on human cognition are yet to be fully elucidated. In light of rich evidence that 17β-estradiol (E2)—the most bioactive estrogen—affects brain structure and function in both sexes^1–6^, it is critical to our understanding of cognition to elucidate mechanisms of interaction between human ovarian milieu and cognitive performance. One approach to accomplishing this is examining differences in cognition across the menstrual cycle.

**Figure 1:**
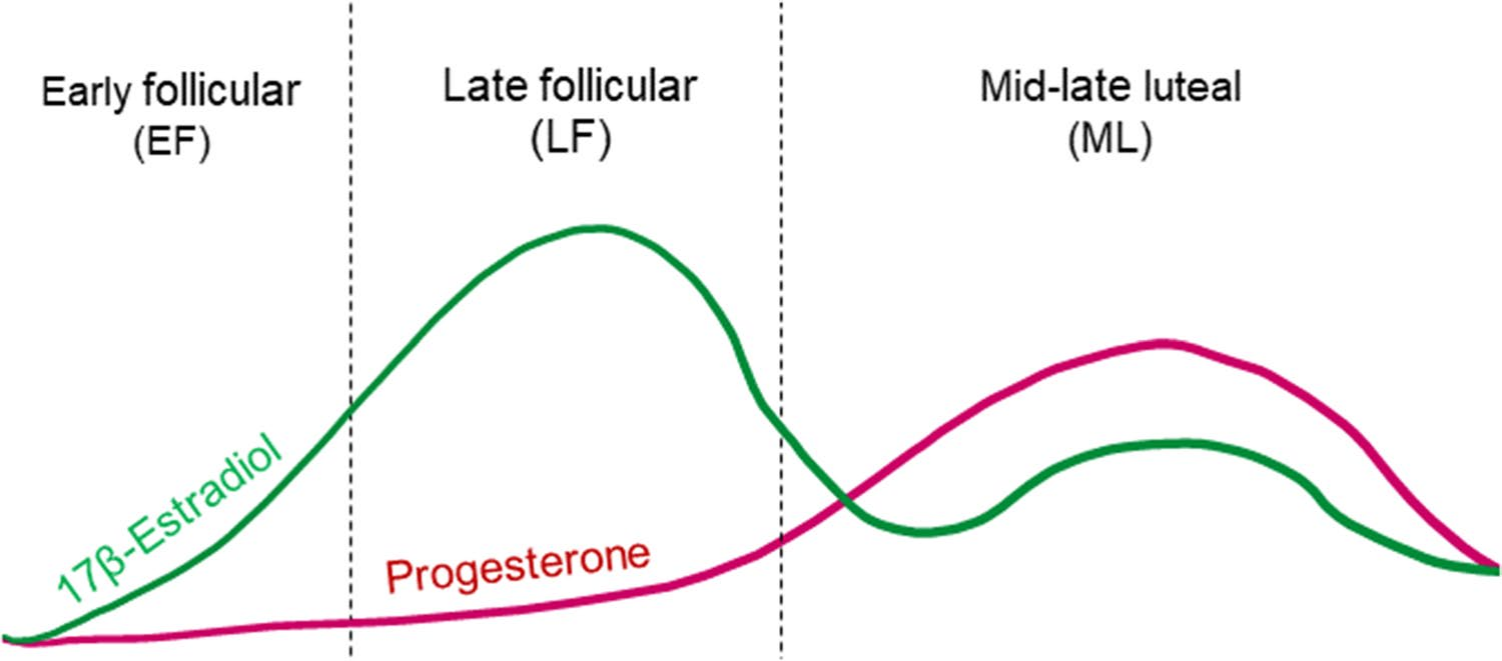
Typical changes in ovarian hormones across the menstrual cycle.

Distinct profiles of endocrine changes across the cycle allow researchers to tease apart effects of different ovarian hormones on cognition. The average menstrual cycle is 29.5 days long, with typical variation ranging from 21 to 35 days^7,8^ and broadly divided into two phases—follicular and luteal—defined by changes in levels of ovarian hormones (Fig. 1). The follicular phase begins with the onset of menses. Its early stage is characterized by low levels of E2 and progesterone. The late follicular or pre-ovulatory phase is characterized by a rise in E2, which reaches its peak shortly before ovulation. The luteal phase follows. During this phase, levels of E2 decrease significantly, settling to moderate levels as progesterone increases during the mid-luteal phase, and decreasing in the late luteal phase as menses approaches^9^. There is, however, considerable variation in hormone levels across the cycle and within the phases. This is especially true of the pre-ovulatory phase^10^.

Although ovarian hormones have widespread effects on the brain^1–6^, one of the most commonly researched brain areas affected by hormonal changes across the menstrual cycle is the hippocampus. Evidence from rodent models suggests that E2 is a key modulator of hippocampal function and associated learning^11^ and memory^1^. Estrogen receptors α and β as well as the G protein-coupled estrogen receptor 1 densely populate the region^12,13^. Through its action on these receptors, E2 has extensive effects on hippocampal dendritic spine density^14^, neurogenesis^15^, cell signaling^1^, and synaptic plasticity^16^. Human studies provide further evidence of E2’s role in hippocampal structure and activity. E2 levels across the menstrual cycle are positively associated with hippocampal grey matter volume^17–20^; activity during affective, visuospatial, and verbal processing^19,21,22^; and functional connectivity with other brain regions^18^. Furthermore, administration of E2 to naturally cycling women in the early follicular phase increases hippocampal activity when the E2 dose elicits an increase within physiological ranges typical of the pre-ovulatory phase^23^.

Converging evidence comes from studies on cognition across the menstrual cycle that use hippocampal-dependent or related tasks^24^. Effects of estradiol on cognition across the menstrual cycle have been demonstrated in verbal memory^25^, verbal fluency^26^, and spatial ability^25–29^. Whereas verbal memory seems to benefit from higher estradiol levels^25^, the opposite is true of mental rotation^26,28^. Furthermore, navigation strategies and ability^30^, mental rotation^29^, and memory for faces^30^ vary across the menstrual cycle in patterns suggestive of estradiol’s effect (e.g., better memory of faces in the high-estradiol, late follicular phase^30^) even in the absence of direct hormone measures, suggesting that even self-reported menstrual cycle phase information can provide clues about effects of ovarian hormones on cognition.

One core cognitive process that is likely sensitive to E2-dependent alterations in hippocampal structure and function is category learning, specifically the learning of exceptions to category rules. Category learning is the ability to extract regularities and dissimilarities across experiences and group common elements into meaningful concepts. Although it engages multiple brain regions^31^, category learning is strongly associated with hippocampal function^32–38^. Learning general patterns in category structure as well as encoding and differentiating exceptional category members (e.g., birds fly; penguins are birds despite being flightless) necessitates episodic memory processes such as relational binding and rapid formation of multi-featured memory representations, all of which are key aspects of hippocampal function^39,40^.

Indeed, studies employing a rule-plus-exception (RPE) category learning task—in which the majority of stimuli can be categorized according to a simple rule, but rare exception items that are more visually similar to the opposing category must be distinctly associated with the correct category—specifically implicate the hippocampus in successful learning^33–35,38^. The hippocampus shows greater activation for exception items in fMRI studies^33^, and diffusion-weighted imaging shows an association between integrity of the hippocampal trisynaptic pathway and exception learning^38^. Hippocampal neural network models of exception learning show convergent evidence^25,41^. Notably, E2 increases potentiation of synaptic transmission within hippocampus, including the trisynaptic pathway^6,32^ which is necessary for exception learning. This suggests that E2-mediated changes to hippocampal function across the menstrual cycle would be reflected in cycle-dependent differences in category learning performance.

**Figure 2:**
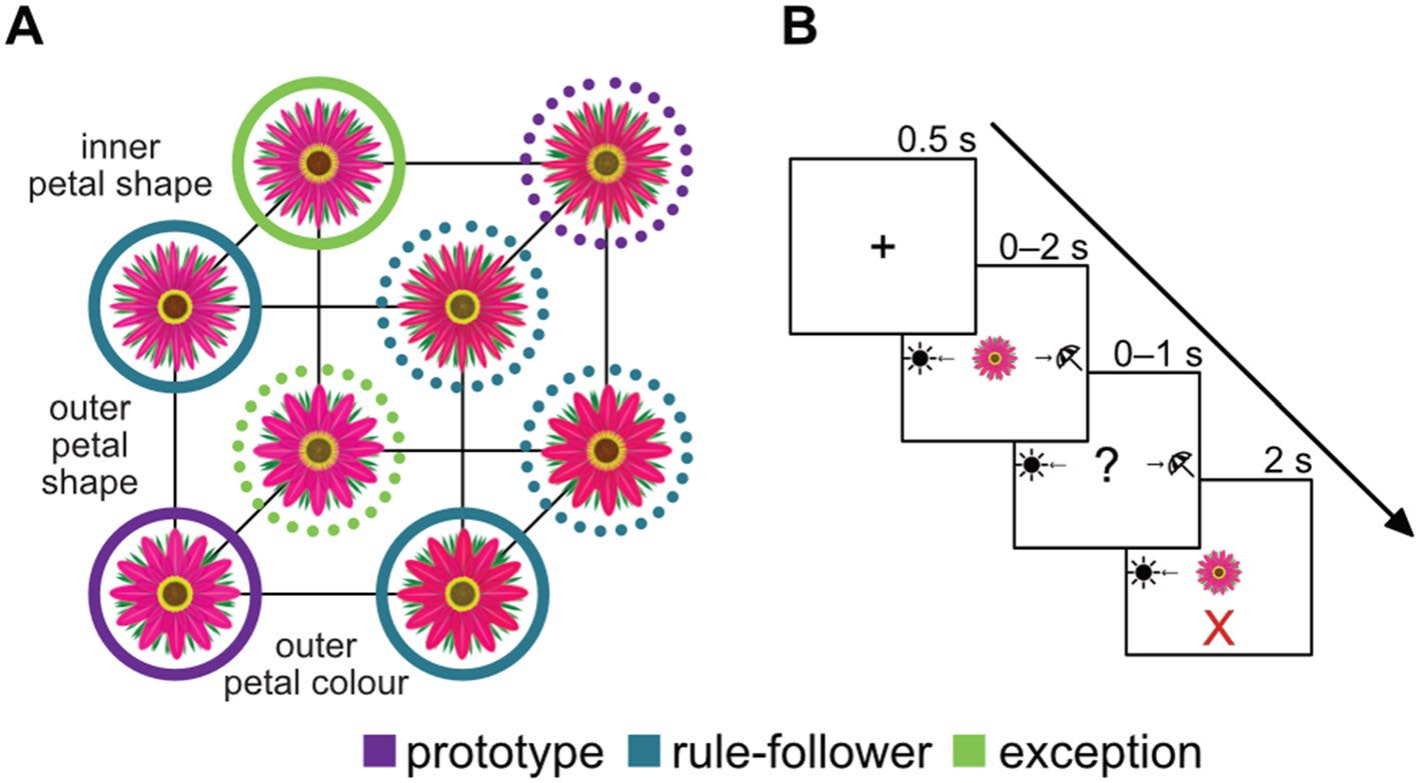
Category structure and experimental trial schematic. (**A**) Flower stimuli consisted of three binary-valued dimensions with categories defined by a rule-plus-exception structure (solid and dotted circles denote category membership). Stimuli were either prototypes (purple; maximally dissimilar across categories), rule-followers (blue; visually similar to their prototype), or exceptions (green; more similar to the prototype of the opposite category, making them more difficult to categorize). The binary diagnostic categories included: shape of outer (pink) petals, shape of inner (yellow) petals, and color of outer petals (more pink vs. more red) (**B**) The task consisted of three learning blocks and a no-feedback test block. Learning trials consisted of fixation (0.5s), presentation of the flower stimulus (2s), a response window allowing the participant to indicate whether a given flower preferred sun or shade (the choice is represented in the figure by sun and umbrella icons on either side of the flower) (1s), and ended with corrective feedback (2s). Learning trials were followed by the test trial.

To test the effect of the menstrual cycle on category learning, we administered an RPE task (Fig. 2) to participants in three stages of the menstrual cycle—early follicular (EF), late follicular/pre-ovulatory (LF/PO), mid/late luteal (ML)—as well as a male group for a low circulating E2 comparison. Given the impact of E2 on the hippocampus and the established role of the hippocampus in RPE learning, we predicted that learning of exceptions would distinctly vary between participants at different points in the menstrual cycle, in accordance with the assumed cycle of E2. Specifically, we predicted that participants in the assumed high-E2, LF/PO phase would show evidence of more efficient learning and outperform participants in the assumed low-E2, EF phase and men.

## Results

### Test performance

To characterize the difference in learning across the menstrual cycle, we calculated a scaled cycle point variable (cycle point = current day of cycle/cycle length) for each participant and modeled its effect on categorization accuracy of the three stimulus types in the test block using generalized additive modeling (GAM). Relative to a baseline linear mixed-effects model with no non-linear effects on cyclepoint, the GAM resulted in a better fit of the data (GAM AIC: − 242.6 vs. linear AIC: − 224.6). Results from the GAM demonstrated a significant overall effect of cycle point (EDF = 1.31, *F*(8) = 13.13,* p* = 0.048) with a distinctly non-linear effect on categorization of exceptions (EDF = 2.93, *F*(8) = 85.17,* p* < 0.001), as presented in Table 1. Bootstrap resampling results confirmed the reliability of this non-linear cycle point effect for exceptions, with 97.7% of bootstrap estimates showing > 1 EDF (EDF_boot_ = 3.86, *p* = 0.02, 95% CI [1.04, 6.14]). Each bootstrap iteration included resampling with one participant excluded and re-fitting the model, suggesting a robust non-linear effect of cycle point on exception learning that was not driven by specific participants. A follow-up analysis examining the effect of each stimulus type separately showed converging results, with a stronger cycle point effect for exceptions (EDF = 2.02, *F*(8) = 0.95,* p* = 0.01) than both rule followers (EDF = 1.25, *F*(8) = 0.44,* p* = 0.05) and prototypes (EDF = 1.22, *F*(8) = 0.39,* p* = 0.07).

**Table 1:** GAM summary.

Parametric terms	Estimate	SE	t-value	*p* value
(Intercept)	0.88	0.02	55.94	< 0.001
Rule-follower	− 0.19	0.02	− 10.59	< 0.001
Exception	− 0.29	0.02	− 15.98	< 0.001

Smooth terms	EDF	Ref.df	F	*p* value
s(cycle point)	1.31	8	13.13	0.048
s(Prototype)	0	8	< 0.001	1
s(Rule-follower)	0	8	< 0.001	0.562
s(Exception)	2.93	8	85.17	< .001

**Figure 3:**
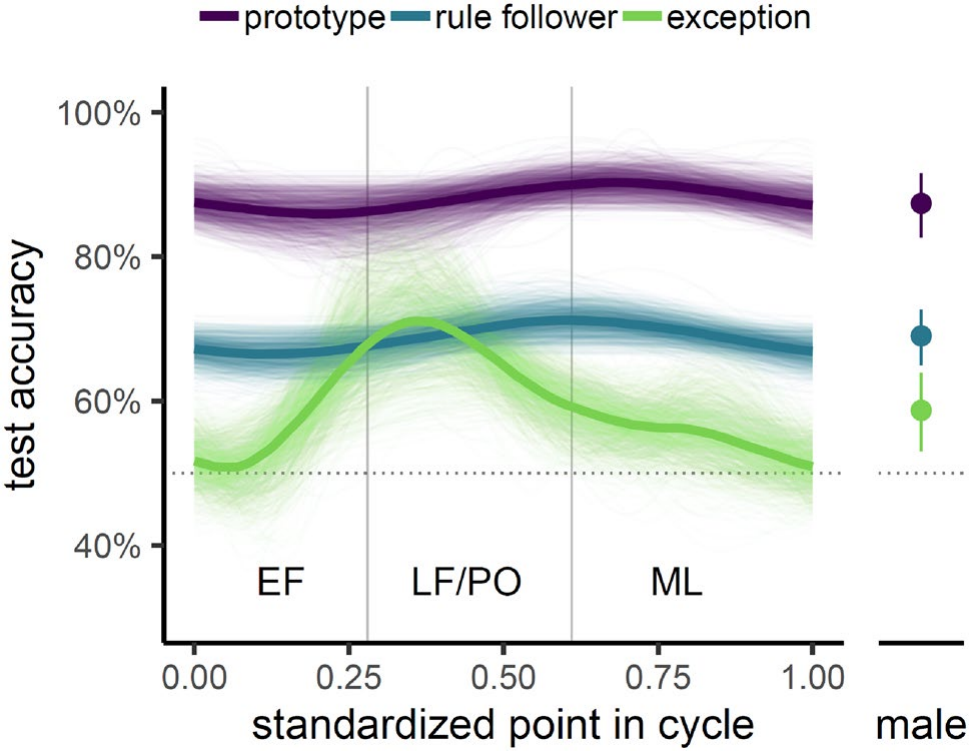
Categorization accuracy in the test block for different stimulus types (prototype—purple, rule follower—blue, exception—green) across standardized points in the menstrual cycle (lines) and in comparison to the male group (points). Median GAM estimates are depicted with thick lines; thin transparent lines depict individual bootstrap resampling GAM estimates. For the male group, average accuracy and 95% confidence intervals are depicted by filled circles and error bars, respectively. See supplemental Fig. S1 for a scatter plot of the raw data.

These results confirm the clear pattern of categorization accuracy across cycle points (Fig. 3); there is a selective increase in categorization accuracy for exceptions across the EF phase that peaks in LF/PO and decreases again in the ML phase. Interestingly, and consistent with our prediction, these cycle-dependent effects in exception learning correspond with typical changes in E2 levels across the menstrual cycle (Fig. 1).

**Figure 4:**
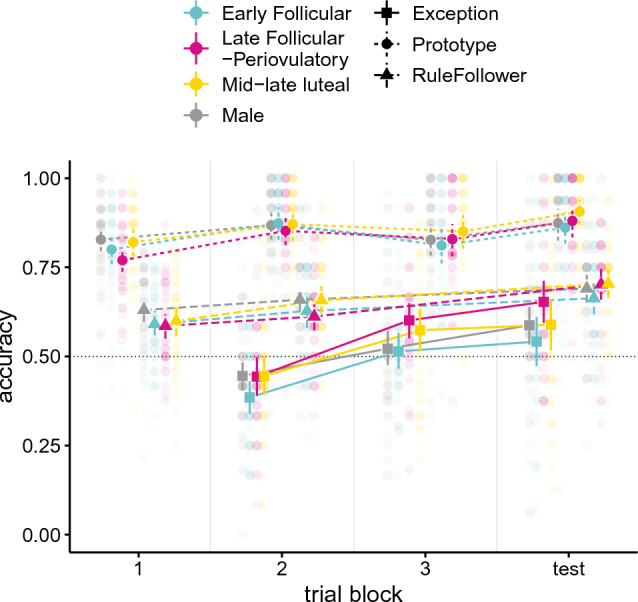
Categorization accuracy across all trial blocks. Average accuracy for stimulus type (prototype—circle, rule-follower—triangle, exception—square) in each learning and test block is depicted separately for the different cycle phase groups (early follicular [EF]—cyan, late follicular/periovulatory [LF/PO]—magenta, mid-late luteal [ML]—yellow) and males (grey). Prototypes and rule-followers appeared in all trials. Exceptions were introduced after a delay, in trial block 2. Error bars represent bootstrapped 95% confidence intervals and transparent points depict individual participant accuracies. The dotted grey line represents chance level (0.5).

We further analyzed categorization accuracy in the test block by discrete groups corresponding to cycle phases (Fig. 4), as is typical in the menstrual cycle and cognition literature^42^. Linear models showed that test block categorization accuracy was higher in the LF/PO group than in the EF group (*β* = − 0.11, *SE* = 0.05, *t*(167) = − 2.31, *p* = 0.02), but that it did not significantly differ from the ML (*β* = − 0.06, *SE* = 0.05, *t*(167) = − 1.33, *p* = 0.18) or male (*β* = − 0.07, *SE* = 0.04, *t*(167) = − 1.47, *p* = 0.14) groups. The EF and ML groups also did not differ in accuracy for exceptions (*β* = 0.05, *SE* = 0.05, *t*(167) = 0.98, *p* = 0.33), and neither did the EF and male groups (*β* = 0.05, *SE* = 0.05, *t*(167) = 1.01, *p* = 0.31) nor the ML and male groups (*β* = − 0.00, *SE* = 0.05, *t*(167) = − 0.37, *p* = 0.97). There were no differences between groups in terms of categorization accuracy for rule-followers or prototypes in the test block (all *p* > 0.05). The analysis of test block accuracy by menstrual cycle phase largely converged with the GAM analysis; however, the more nuanced changes in exception learning with notable dynamics occurring within phases were better captured by the GAM analysis.

### Learning performance

Further, we analyzed performance across trial blocks to assess the process of learning exceptions by group. Results of linear mixed-effects models indicate that the EF group had lower accuracy for exceptions across learning blocks (blocks 2 and 3) relative to the LF/PO (*β* = 0.07, *p* = 0.01, 95% CI [0.02, 0.12]) and ML groups (*β* = 0.06, *p* = *0.0*3, 95% CI [0.01, 0.11]), but not relative to the male group (*β* = 0.03, *p* = 0.15, 95% CI [− 0.01, 0.08]). The LF/PO and ML groups did not differ in accuracy for exceptions across learning blocks (*β* = − 0.02, *p* = 0.53, 95% CI [-0.07, 0.03]), nor did the LF/PO and male groups (*β* = − 0.04, *p* = 0.12, 95% CI [− 0.09, 0.01]) or the ML and male groups (*β* = − 0.02, *p* = 0.36, 95% CI [− 0.07, 0.03]). There were no differences between groups in terms of accuracy for rule-followers or prototypes across learning blocks (all *p* > 0.05). A follow-up generalized linear model analysis of learning slopes after exceptions were introduced (i.e., difference scores between blocks 3 and 2) indicated that categorization accuracy of the LF/PO group for exceptions improved more quickly than that of men (*β* = − 0.09, *SE* = 0.04, *t*(164) = − 2.19, *p* = 0.03, *d* = 0.39), but at a similar rate as the EF (*β* = − 0.04, *SE* = 0.04, *t*(164) = − 0.92, *p* = 0.36) and ML (*β* = − 0.03, *SE* = 0.04, *t*(164) = − 0.74, *p* = 0.46) groups. The male group did not significantly differ from the EF (*β* = 0.04, *SE* = 0.04, *t*(164) = 1.18, *p* = 0.23) and ML (*β* = 0.05, *SE* = 0.04, *t*(164) = 1.38, *p* = 0.16) groups.

## Discussion

This is the first study to examine category learning across the menstrual cycle. We find that the menstrual cycle affects learning of exceptions in a distinct way that parallels the typical E2 cycle. Categorization accuracy for exceptions increases steadily across the EF phase, peaking in LF/PO, and decreasing again over ML, with participants in the assumed high-E2, LF/PO, outperforming those in the assumed low-E2, EF, phase. Over the course of exception learning, participants in the LF/PO and ML (moderate E2) phases both outperform EF participants, and those in the LF/PO phase learn to categorize exceptions more quickly than the male group.

That participants in the LF/PO phase show improved performance relative to those in the EF phase and the male group, with participants in the ML phase also showing better performance than EF participants during learning, suggests a role of E2 in facilitating exception learning. Our finding are in line with evidence that E2 supports a range of learning tasks in rodent models^1^ and human studies showing that differences in hippocampus-dependent tasks vary by ovarian milieu^24–26,29^. Furthermore, performance on associative memory tasks—which, like category learning, require rapid relational binding—is also positively associated with E2 levels^43^.

We hypothesize that the RPE task is likely affected by E2’s action on the hippocampal subfields implicated in pattern separation and completion. These processes are key to successful exception learning as such learning requires generalization with and separation from previously learned category exemplars. Presumably, CA1-dependent formation of rule-based category representations^37^ takes place during the initial stages of the task. Once exceptions are introduced, the mismatch signaling of these stimuli to previously stored category items triggers pattern-separation processes in dentate gyrus and CA3 to distinctly encode exceptions and their association with the correct category^33,36,38^. Notably, E2 increases synaptic density in CA1^1^; increases long-term potentiation at CA3–CA1 synapses^6^; and potentiates synaptic transmission in CA1, CA3, and dentate gyrus, with the greatest magnitude of potentiation observed in CA3^44^. Our findings converge with these established neurobiological impacts of E2 on hippocampus.

The fact that exception categorization performance declines in participants in the ML phase of the cycle is in line with research on interacting effects of E2 and progesterone in the hippocampus. Rodent models suggest that increases in E2 across the estrous cycle are positively associated with hippocampal spinogenesis, while progesterone inhibits this effect^45^. Similarly, progesterone attenuates the positive effect of E2 on hippocampal subfield volume in humans^46^. Seemingly in line with these brain data, a behavioural effect similar to ours is found in studies on the menstrual cycle effects on verbal memory, a task with demonstrated hippocampal involvement^47^. Verbal memory performance is improved and positively associated with estradiol during the LF phase^25,48^ but it declines in the ML phase^25^, at times to the point of not being significantly different relative to the EF phase^48,49^. These results mirror ours, suggesting either a dose-dependent E2 effect or an effect of E2 counteracted by progesterone.

A major strength of the current study is its sampling of participants at various phases of the menstrual cycle. Most studies on cognition across the menstrual cycle only include two phases^42^ and may thus be less sensitive to effects of hormonal changes. Comparison of the EF and LF/PO phases is especially relevant as it allows examination of low and high E2 periods while levels of progesterone are low. Notably, most studies on cognition across the menstrual cycle (e.g.,^50–54^) compare the EF phase to the ML phase, when E2 is moderate and progesterone is high, thus introducing a possible confounding factor. By sampling from all phases and creating a continuous standardized cycle variable, we were able to characterize learning behavior as it evolves across the cycle.

A further strength of the current approach is the use of non-linear methods to examine changes across the menstrual cycle. Analyzing menstrual cycle phases as discrete categories may obscure variance within the phases or mask some of the differences in performance that emerge across the cycle. In contrast, the GAM approach allows us to identify continuous changes in category learning across the cycle. Beyond knowing that participants in the LF/PO phase outperform those in the EF phase, we can observe a nuanced rise and decline in categorization accuracy across the cycle that mirrors expected cycle-dependent changes in E2 levels. This is especially informative as there are significant individual differences in hormonal changes across the menstrual cycle, with the LF/PO phase being the most variable^10^. The current GAM approach allows us to map the behavioural reflection of that variance in a larger sample, accounting for individual variance through bootstrap resampling. Non-linear approaches in future studies may facilitate a deeper understanding of the effects of this variance in phase timing on cognition.

The main limitation of the current study is its lack of direct hormone measures. We therefore cannot be certain that the determined cycle phases correspond to assumed levels of E2 and progesterone, and we cannot account for other hormones that vary across the menstrual cycle such as the follicle-stimulating hormone and luteinizing hormone. However, prior literature suggests that self-reported data of menstrual cycle phase aligns with serum hormone levels^25^, and the average days of cycle per group in the current study are akin to those reported in literature on cognition across the three menstrual phases with confirmed hormone levels^55^. It is also worth noting that our data-driven GAM approach, which utilized cyclical basis functions, is agnostic to when deviations in learning performance across the cycle should emerge if present. That the observed changes in exception learning followed typical cycle-dependent E2 levels offers compelling evidence for a potential association. Nonetheless, future work would benefit from inclusion of blood or saliva hormone assays.

The current RPE task potentially offers an avenue for characterizing the neural mechanisms of ovarian hormones’ effects on category learning through neuroimaging methods like fMRI. As administration of E2 to women in the early follicular phase increases hippocampal activity^23^ and E2 increases hippocampal activation during the pre-ovulatory phase in naturally cycling women^19^, we expect to see a link between E2-driven effects on hippocampal function and behavioral evidence of more efficient categorization of exception learning during the LF/PO phase. Follow-up fMRI studies should also examine brain regions beyond the hippocampus, as ovarian hormones have whole-brain functional effects^56^ and hippocampal connectivity to the frontal and parietal cortices—two regions heavily implicated in category learning^31,57^—varies across the menstrual cycle^18,58^, along with activation of other areas relevant to category learning such as the striatum^55,59,60^.

Overall, this work provides novel evidence of the role of the menstrual cycle in learning exceptions to category rules, adding a key new factor to the multi-faceted literature on category learning and further elucidating the possible effects of ovarian hormones on human cognition. We demonstrate the value of using non-linear methods to study continuous changes in cognition across the menstrual cycle, and the cycle-phase-dependent changes we observe provide impetus for increased inclusion of menstrual cycle data in cognitive neuroscience research. For researchers interested in hippocampal function, E2 levels could provide a targeted manipulation to explore how subtle, sub-field-level hippocampal changes influence the neural mechanisms underlying any number of cognitive tasks.

## Methods

### Participants

Participants were recruited through the Prolific online recruiting platform and were prescreened for age (18–35), normal or corrected-to-normal vision, and English fluency. Our target sample size was based on prior studies that have employed RPE tasks similar to the one used in the current study^35,38^. As such, we targeted 40 participants per group (i.e., 160 participants total). A total of 260 participants completed the study. Participants were excluded if they reported any of the following: irregular menstrual cycles (n = 15), use of hormonal birth control (n = 62), use of hormone replacement therapy (n = 1), a history of neusrological conditions that may affect cognitive performance (e.g., stroke, traumatic brain injury; n = 2), or if they had an accuracy of under 0.75 for any stimulus type in at least one trial block of the RPE task or if over 20% of their reaction times fell outside of the 0.15–2s range (n = 9).

After exclusions, 171 participants remained for analysis (age: 29.59 ± 5.05 years, education: 15.89 ± 3.41 years). The majority (79%) of participants tracked their cycles using an app or a calendar. There were 39 participants in the EF phase, 40 in the LF/PO phase, 39 in the ML phase, and 53 in the male group. Average menstrual cycle length was 27.87 ± 5.03. On average, the EF group was on day 4 ± 3.58 of their cycle; the LF/PO group, on day 13.1 ± 3.25; and the ML group, on day 21.5 ± 3.42.

### Procedure

After providing informed consent, participants completed a category learning task followed by a questionnaire assessing demographic and health-related information, including menstrual cycle information. Participants received monetary compensation for participation in the study. All research protocols used in the study were approved by the University of Toronto Research Ethics Board. All experimental procedures were conducted in accordance with the University of Toronto Research Ethics Board and all participants provided informed consent.

### Category learning task

Participants completed a RPE categorization task^35,61^ consisting of three learning blocks and a no-feedback test block. Throughout the experiment, participants viewed 10 images of flowers with three binary-valued diagnostic dimensions and one non-diagnostic dimension: outer petal colour, outer petal shape, inner petal shape, and central disc colour (Fig. 2A). As the central disc color was determined to be least salient in a norming study, this dimension was chosen to be nondiagnostic, varying randomly between stimuli. Flowers belonged to either a “prefers shade” or “prefers sun” category. Flower stimuli were prototypes (maximally dissimilar across categories), rule-followers (more similar to their category prototype than to the prototype of the opposite category), or exceptions (more similar to the prototype of the opposite category). There were four prototypes (two per category for each value of the non-diagnostic feature), four rule-followers (two per category with random non-diagnostic feature values), and two exceptions (one per category, also with random non-diagnostic feature values), for a total of 10 stimuli split evenly between the two categories. Participants completed three learning blocks of 48 trials each. In each learning trial, participants were shown a flower image for up to 2s and used the keyboard to indicate whether it preferred sun or shade. They were then given corrective feedback on the accuracy of their response for 2s (Fig. 2B). The distribution of stimulus type (prototype, rule-follower, and exception) varied across the learning blocks. Learning block 1 consisted only of prototypes and rule-followers (24 trials each), learning block 2 included all three types (24 prototypes, 12 rule-followers, and 12 exceptions), and learning block 3 included only prototypes and exceptions (24 trials each). This delayed-exception learning sequence has been shown to improve exception learning, potentially through engagement of hippocampus-based encoding computations^35^. Participants then completed a no-feedback test block with 48 trials evenly split between the three stimulus types. In these trials, a flower was shown for up to 2s and a category response was made, but no feedback was provided.

### Menstrual cycle phase determination

To account for variability in length of menstrual cycles (21–35 days^8^), menstrual cycle phases were determined according to each participant’s self-reported cycle length and current day of cycle. The phases, with predicted hormone levels, were as follows: EF—approximately 1–7 days after menses onset, predicted low E2 and progesterone; LF/PO—approximately 8–17 days after menses onset, predicted high E2 and low progesterone; ML – approximately 1–11 days prior to menses onset, predicted moderate E2 and high progesterone.

### Statistical analyses

All statistical analyses were completed in *R* (version 4.0.3). To analyze test performance after learning, we fit a generalized additive model (GAM; *gamm4* package) with a scaled variable denoting participants’ current point in the menstrual cycle (cycle point = current day of cycle / cycle length) as a predictor of categorization accuracy in the test block. Creating this standardized variable allowed us to examine if the pattern of categorization accuracy across the standardized cycle corresponded to assumed changes in ovarian hormone levels across the menstrual cycle (Fig. 1). By tracking performance at multiple points in the cycle in a continuous fashion, we were able to examine behavioural variability within menstrual cycle phases in a way that is not possible when using traditional categorical analyses. The GAM included smoothing on the cycle point predictor with a cyclic cubic spline basis function, an interaction with stimulus type (exception, rule-follower, prototype), and random intercepts for participants. We evaluated the reliability of the model through bootstrap resampling at the participant level with 1000 iterations and report the median estimated degrees of freedom of the resamples (EDF_boot_) with 95% confidence intervals. EDFs in the context of GAM provide a measure of non-linearity, with an EDF of 1 indicating a linear effect and values higher than 1 indicating non-linearity. The bootstrap resampling at the participant level allowed us to make sure the effect was not driven by specific participants. Further, we fit a generalized linear model predicting test accuracy by group (EF, LF/PO, ML, male) based on predefined phases of the menstrual cycle, as is typically done in menstrual cycle and cognition literature^42^.

Categorization accuracy was analyzed across learning blocks using generalized linear mixed-effects models predicting categorization accuracy by participant group and stimulus type, and with participants included as random effects using the *lme4* and *lmerTest* packages. This analysis was followed by a generalized linear model examination of learning slopes (i.e., difference scores between blocks) by group for exceptions.

### Supplementary Information

Supplementary Figure S1.

## Data Availability

Data and materials can be found on OSF at https://osf.io/9nwvz.
