# Supplementary figures and images for: Learning exceptions to category rules varies across the menstrual cycle
Source: Sci Rep. 2023 Dec 12;13:21999. doi: 10.1038/s41598-023-48628-x (PMC10713535; doi:10.1038/s41598-023-48628-x)

# Prototype

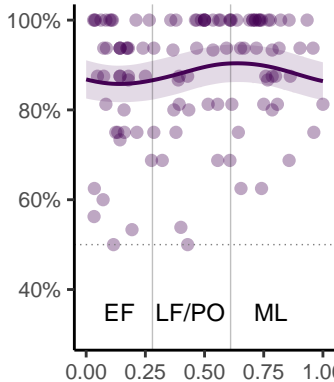

# RuleFollower

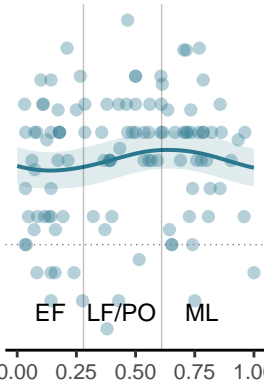

# Exception

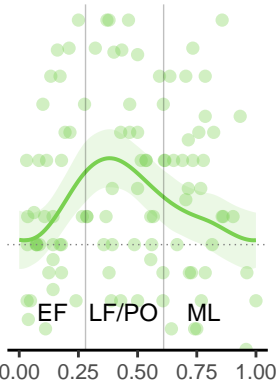

Supplement: Supplementary file 1 — Supplementary Figure S1. [file 41598_2023_48628_MOESM1_ESM.pdf]
